# A cancer care desert: living in between the urban and rural and the case for defining semirural regions

**DOI:** 10.3389/fonc.2023.1204821

**Published:** 2023-05-22

**Authors:** Laurel J. Finster, Sarah J. Salvy, Robert W. Haile

**Affiliations:** Cancer Research Center for Health Equity, Division of Medical Oncology, Cedars-Sinai Medical Center, Los Angeles, CA, United States

**Keywords:** cancer prevention and control, healthcare disparities, semirural, rural health, health equity (MeSH)

## Introduction

The intersection of social, structural, and geographic determinants can have profound effects on cancer prevention, early detection, treatment, and survivorship care (1). Cancer disparities become more pronounced in rural regions as residents face lower access to healthcare, health services, and insurance coverage compared to their urban counterparts (2). Lower levels of education and economic instability, paired with higher levels of poverty and unemployment in these regions can lead to bleak health outcomes (3–5). Fortunately, the field of rural health is maturing and funding is now being allocated to these populations in need (6). However, this binary approach has largely excluded individuals who reside in between “urban” and “rural” regions, leaving semirural populations to fall between the cracks while disparities worsen.

## Defining urban versus rural

The U.S. Census Bureau has been reporting urban and rural statistics for over a century, and although they recognize that an urban/rural spectrum exists (7), the classification of these geographies remains subjective, with many funding agencies using varying indicators of classifications. Current classifications include: USDA Rural-Urban Continuum Codes (RUCC), Rural-Urban Commuting Area (RUCA), Frontier and Remote (FAR) Census Urban Rural Indicator Code (URIC). It is estimated that 55% of Americans (roughly 175 million) live in what has traditionally been referred to as “suburbs” or “small metro counties” (8). Suburbs have historically lagged behind urban counterparts in growth in education, income and home values, and more recently, suburbs have been experiencing a growing population of older adults, and an increase in poverty and unemployment (9, 10).

These regions often mirror the economic and social trends of traditional suburbs yet face many of the same challenges of rural regions as they serve both rural and urban populations. Without a formal definition, these “in between” communities continue to be excluded from critical funding, policymaking, and resource allocations. For example, regions across the U.S. such as Salinas, an urban area located along the eastern limits of the Monterey Bay Area, or the Antelope Valley (AV) in Los Angeles County, are semirural communities living at the intersection of urban and rural. Salinas, which has a population of 163,542, is one of the most productive agricultural regions in California yet is considered an urban region. The AV, which is home to roughly 437,860 people, constitutes the western tip of the Mojave Desert and has the highest percentage of people who live in rural tracts in all of Los Angeles County, yet is also considered urban^1^ (11–13). Semirural communities like these often struggle with adequate public health infrastructure, leading to lack of preventative services and health professional shortages in primary care, dental care, and mental health (11, 14–16).

Due to their urban classification, these regions are not eligible for many rural development programs in the areas of health, housing, and other essential services despite serving as a healthcare hub for many bordering rural communtiites (17). For example, they would not qualify for the Federal Office of Rural Health Policy grant programs or the Rural Health Clinics Program (11), despite facing many of the same challenges that traditional rural communities face. And when it comes to support for cancer care, major gaps exist. Although rural areas typically see a lower incidence of most cancer types, mortality rates are often higher (18, 19), suggesting a later stage of diagnosis, lower quality care, lower rates of preventive screening, and/or other barriers that may be unique to rural populations (17, 20). There is not a clear understanding of where semirural populations fall along this cancer continuum, or the types of policies, programs, or funding mechanisms needed to support them.

Below we highlight three themes identified that support the need to uniquely classify semirural populations. These include geographic barriers (e.g., public and private transportation), opportunities for healthy behaviors to reduce disease risk (e.g., physical activity, access to nutritious food), and access to healthcare (e.g., cancer prevention and early detection services).

*Geographic barriers in dispersed and low-density regions*. Adequate transportation is a social determinant discussed at length in the literature in terms of access to nutritious food, healthcare and social services (21), employment, and educational opportunities (22, 23). It is also well documented that people with cancer living in rural areas face more transportation challenges, which can be associated with delayed follow-up after abnormal screenings, decreased access to specialized oncology care, and lower treatment adherence to treatment recommendations (20, 24) Dispersed and low-density regions face challenges such as long distances and lack of sidewalks, discouraging walkability, and in turn fortifying reliance on private automobiles and/or public transportation such as rail and bus services. People living in rurality are dependent on public transport, yet less than 10% of federal funding for public transportation goes to rural areas, with no distinction for those living in between rural and urban regions (20). Even when public transport is offered in a community, semirural regions can experience additional geographic challenges in these services. Public transport network can be underutilized due to poorly designed transit stops that are not easily accessible (25). Downstream, this discourages people from utilizing public transport and limits access to critical health services.

*Opportunities to engage in healthy living.* The relationship between food access, poverty, and transportation is complex. Rural and semirural residents are particularly isolated, with even greater limitation in access to opportunities to engage in physical activity and nutritious and low-cost foods. Many communities across the geographic spectrum can experience what is known as a ‘food desert’, where the availability, accessibility, and affordability of nutrient dense foods are limited in favor of processed, shelf-stable, and energy dense foods that contain added sugar, fats, and refined grains (26). However, rural and semirural communities often face a double burden of living in food deserts and food assistance deserts (27), both of which can result in poor health outcomes and higher disease risk (28–31).

Further, walkable, bikeable, and transit-oriented communities are associated with adherence to physical activity guidelines, which is key to chronic disease prevention and management (32). Importantly, many residents in semirural communities have to commute multiple hours daily to make a livable wage, which also limits their opportunities to engage in other healthy behaviors like cooking or exercising (14). Research suggests that lack of time is one of the most common reasons people give for not engaging in healthful behaviors, and time constraints are linked to poorer physical and mental health. Yet, despite this, time as a social determinant of health disparities remains largely unexplored and may be particularly relevant for semirural communities (33).

*Access to Care.* Semirural communities often experience a shortage of adequate healthcare and health information. At the provider level, only one-tenth of physicians practice in these rural areas while one-fifth of the US population resides in these same areas (20). Due to a lack in defining semirural regions, we do not know how many people are considered to live “in between” rural and urban areas. We do know, however, that rural and semirural towns often lack the educational and cultural opportunities, and population size to attract and support medical students, residents, and specialty practices (20, 29). Consequently, there are only 40 specialists per 10,000 people in rural areas compared to 134 specialists per 10,000 people in urban areas (29). In oncologic specialty care, only 3% of medical oncologists practice in rural areas and 70% of counties in the US do not have a resident medical oncologist at all (30). At the system level, residents in more isolated areas have been found to travel nearly three times longer than urban residents to receive cancer treatments such as radiation (20, 30, 31). Essentially, this region and other regions around the US have become cancer care deserts, without adequate support or funding to support the needs of its population.

## Discussion

**Figure 1:**
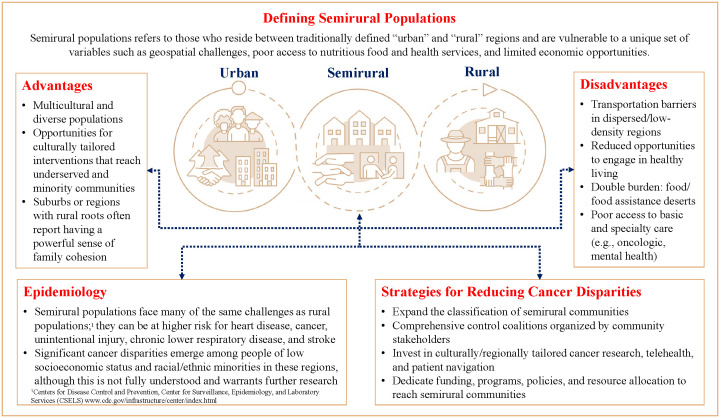
Key considerations for defining semirural regions to address cancer disparities.

Social, structural, and geographic determinants influence the ability to live a healthy life, including access to lifesaving cancer prevention and early detection services. Similar to populations living in rurality, those who reside in between “urban” and “rural” regions are vulnerable to a unique set of variables such as transportation challenges, poor access to nutritious food and health services, and limited economic opportunities. Although the field of rural health is gaining traction, semirural communities are not well defined and often fall between the cracks, widening disparities among people living in poverty and racial and ethnic minorities The results point to significant cancer disparities, particularly among people of low socioeconomic statues and racial and ethnic minorities that warrant further investigation. However, there are region-specific characteristics that can serve as protective factors to promote engagement in healthy living in semirural communities, which can have downstream effects on improving cancer and other health disparities across the continuum (**Figure 1**).

### Protective factors

Semirural communities are complex, ingenious, and crucial to the culture of a nation. There are region-specific characteristics that can serve as protective factors to promote engagement in healthy behaviors in semirural communities. At the community level, these regions can be diverse and multicultural. This multicultural diversity fosters the opportunity for culturally tailored interventions that meet the unique needs of underserved and minority communtities (34). Additionally, suburbs or regions with rural roots often report having a powerful sense of family cohesion, which lends itself to interpersonal interventions to promote healthy living. Strong family cohesion can serve as a protective factor in psychological distress and adolescent development, all of which impact overall health and wellness (34–36). Combined, culturally tailored interventions and strong family support have proven effective at promoting healthy living, timely care, and increased knowledge of disease by participants (37).

### Recommendations for future research and potential implementation strategies

Although a unified strategy is not in place to address the needs of semirural communities, work can be done at multiple levels to improve health equity of these regions. At the grassroots level, community organizations can organize comprehensive control coalitions that focus on cancer prevention, education, screening, access to care, support for cancer survivors, and overall healthy living (28).

At the provider level, advances in telehealth and patient navigation are being made to reach communities with access barriers. Hull and colleagues (2022) highlight that the COVID-19 pandemic forced many health systems to ramp up their telehealth services and they predict that telehealth with remain a critical part of the health system (38). Wercholuk et al. highlight that in cancer care, telehealth interventions can reduce patient travel burden, increase access to specialty oncologic care, and offer patient navigation programs that connect patients to local resources such as free or subsidized non-emergency medical transportation (24). Patient navigation programs have been shown to improve cancer outcomes, particularly among racial and ethnic minorities, and those with low socioeconomic status (39, 40). Patient navigation programs that utilize telehealth could continue to be a valuable tool for semirural communities. However, Hull et al. discuss tradeoffs between convenience and quality when telehealth replaces in-person visits and warns against potential pitfalls of telemedicine that may lead to missed or inaccurate diagnoses. Pairing telehealth with digital diagnostic tools and creating standards for telehealth may be particularly important for remote cancer care (38).

At the federal level, the Biden Administration signed an executive order in 2021 on “Advancing Racial Equity and Support for Underserved Communities” to expand the classification of disadvantaged communities. A set of tools and policies are being developed to address the needs of populations that share “a particular characteristic, as well as geographic communities, that have been systematically denied a full opportunity to participate in aspects of economic, social, and civic life, including the impartial treatment of all individuals, including individuals who belong to underserved communities that have been denied such treatment, such as Black, Latino, and Indigenous and Native American persons, Asian Americans and Pacific Islanders and other persons of color; members of religious minorities; lesbian, gay, bisexual, transgender, and queer (LGBTQ+) persons; persons with disabilities; persons who live in rural areas; and persons otherwise adversely affected by persistent poverty or inequality.” This is particularly good news for communities that have been historically unseen. Further, early in 2022, President Biden announced a reignition of the Cancer Moonshot, highlighting new goals: to reduce the cancer death rate by half within 25 years and improve the lives of people with cancer and cancer survivors. The next phase of the Cancer Moonshot Initiative includes The NCI Telehealth Research Centers of Excellence (TRACE) program that aims to determine whether the use of telehealth can improve cancer-related care and outcomes across the cancer control continuum.

Lastly, several other structural determinants of health should be examined to determine their impact on health behaviors across the cancer continuum, including structural racism, mass incarceration, and the Affordable Care Act and its implications for health-care equity. In addition, factors that may be unique to semirural populations and warrant further investigation include populations who are unhoused or sheltered homeless, mental and behavioral health, smoking, obesity, and challenges at the family and community level.

Just as the case has been made to invest in rural areas (18, 41), semirural areas require investment in region specific research, resources, and policies. These important multilevel initiatives to reach underserved communities, paired with an expanded classification of semirural communities and further examination of protective factors, stands to significantly close the gap on the disproportionate cancer disparities experienced by millions living in cancer care deserts across the United States.

## Author contributions

LF wrote the first draft of the manuscript. LF, SS and RH contributed to manuscript revision and approved the submitted version. All authors contributed to the article and approved the submitted version.

## Footnotes

1 Census 2010 definition of rural and urban (https://www.census.gov/programs-surveys/geography/guidance/geo-areas/urban-rural/2010-urban-rural.html). Urban areas are those inside urbanized areas (50,000 or more people) and urban clusters (at least 2,500 and less than 50,000 people).  “Rural” encompasses all population, housing, and territory not included within these urban areas.

## Supplementary material

Supplement A An example of a semirural region in the Antelope Valley in Los Angeles County, California. When looking at County level data (right), Los Angeles County is classified as a urban region. When looking closer by census tracts (left), the geographic nuances of the semirural region of Antelope Valley reveal a rural serving suburb about 60 miles from the urban center of Los Angeles.
