# Supplementary material for: A cancer care desert: living in between the urban and rural and the case for defining semirural regions
Source: Front Oncol. 2023 May 22;13:1204821. doi: 10.3389/fonc.2023.1204821 (PMC10241065; doi:10.3389/fonc.2023.1204821)
Supplement: Supplement A — An example of a semirural region in the Antelope Valley in Los Angeles County, California. When looking at County level data (right), Los Angeles County is classified as a urban region. When looking closer by census tracts (left), the geographic nuances of the semirural region of Antelope Valley reveal a rural serving suburb about 60 miles from the urban center of Los Angeles. [file Image_1.pdf]

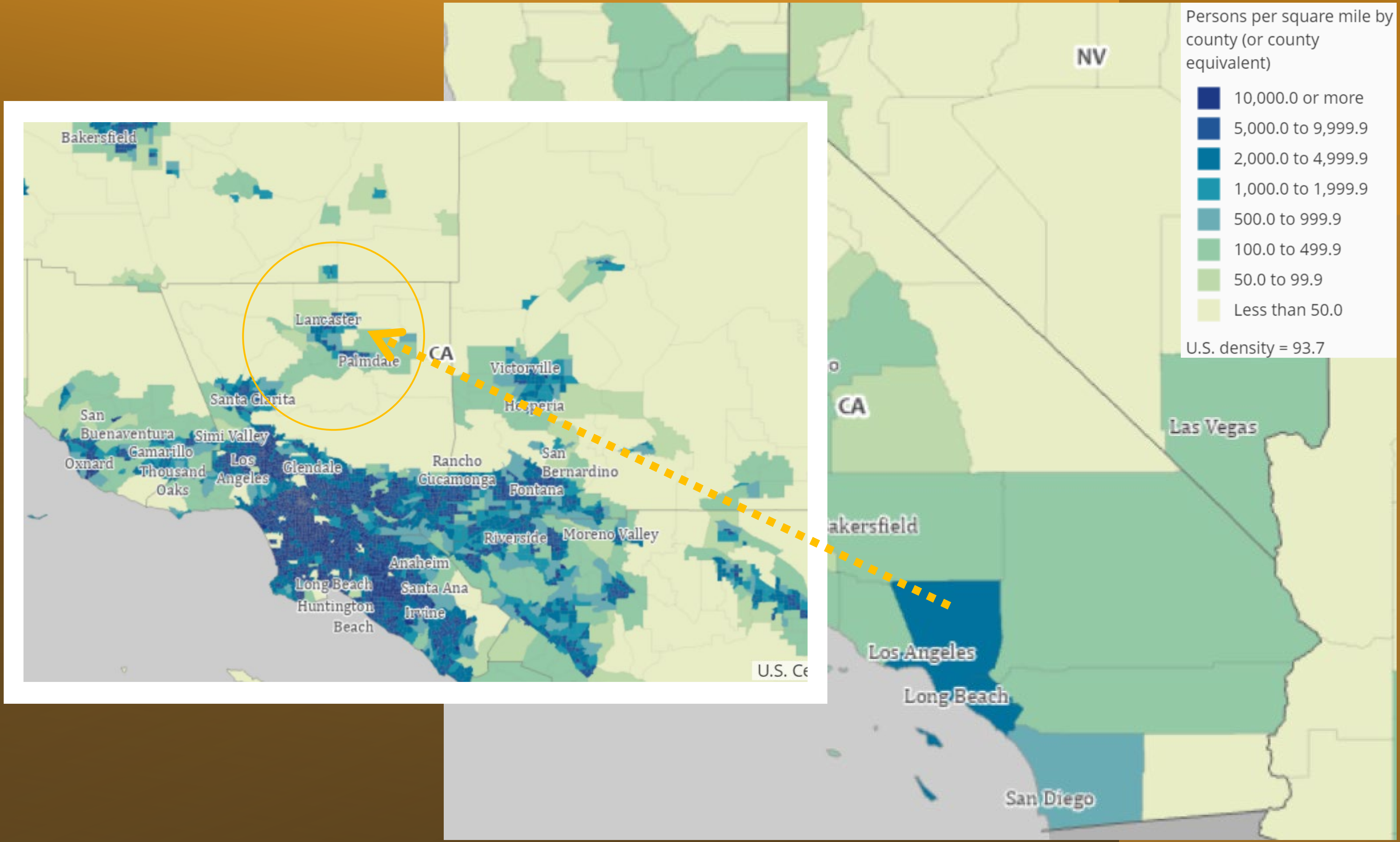

Data from 2020 U.S. Census Bureau, 2020 Census Demographic Data Map Viewer, data.census.gov
